# A clinical study comparing polymer and gold fiducials for prostate cancer radiotherapy

**DOI:** 10.3389/fonc.2022.1023288

**Published:** 2023-02-01

**Authors:** Daryl Lim Joon, Colleen Berry, Benjamin Harris, Mark Tacey, Drew Smith, Nathan Lawrentschuk, Michal Elisabeth Schneider, Olivia Fraser, Megan Hall, Michael Chao, Farshad Foroudi, Trish Jenkins, David Angus, Morikatsu Wada, Shomik Sengupta, Vincent Khoo

**Affiliations:** ^1^ Radiation Oncology Department, Olivia Newton-John Cancer Centre, Heidelberg, VIC, Australia; ^2^ Office of Research, The Northern Hospital, Epping, VIC, Australia; ^3^ Melbourne School of Population and Global Health, Faculty of Medicine, Dentistry and Health Sciences, University of Melbourne, Carlton, VIC, Australia; ^4^ Austin Health, University of Melbourne, Melbourne, VIC, Australia; ^5^ Department of Medical Imaging and Radiation Sciences, Monash University, Melbourne, VIC, Australia; ^6^ Department of Clinical Oncology, Royal Marsden Hospital, London, United Kingdom

**Keywords:** radiotherapy, prostate cancer, polymer fiducials, image guided radiotherapy, verification

## Abstract

**Introduction:**

Image guidance with gold fiducials improves outcomes of prostate radiotherapy. However, gold produces artefact on CT imaging, interfering with contouring and verification. The purpose of this study was to compare polymer to standard gold fiducials using radiotherapy imaging modalities to assess the visibility and artefact.

**Methods:**

Twenty eight patients with locally advanced prostate cancer were enrolled, half had three polymer fiducials implanted into the prostate and half underwent insertion of gold fiducials. Patients were imaged with CT, T2 weighted MRI, cone-beam CT (CBCT) and planar KV images. Fiducials were scored for visibility and assessed for CT artefact in surrounding prostate tissue. The artefact was quantified from Hounsfield number histograms and separated into percentile ranges and proportion of voxels in HU normal tissue range of a 2cm sphere surrounding the fiducial.

**Results:**

Gold and polymer fiducials were sufficiently visible for CT and CBCT verification. The gold fiducials could be visualized well on KV planar imaging; however, the polymer markers were obscured by pelvic bones. Neither polymer nor gold fiducials could be visualized on MRI. The polymer fiducial produced less artefact than gold on CT, having less voxel spread for the HU percentile ranges and a greater proportion of voxels in the normal tissue range.

**Conclusions:**

Polymer fiducials are a more suitable fiducial than gold for CT/CBCT in prostate cancer radiotherapy, demonstrating minimal artefact and good visibility on CT. However, they were not well seen on MRI or KV imaging and thus not suitable for co-registration or planar KV verification.

## Introduction

Prostate cancer is one of the most commonly detected cancers in men and a leading cause of deaths (1). Approximately 10-20% of patients diagnosed with prostate cancer will have locally advanced disease. Randomized studies have shown significant improvements in outcome for locally advanced prostate cancer using higher radiation doses (2–6) and adjuvant androgen deprivation therapy (7–10).

The therapeutic ratio is further improved with precision radiotherapy techniques such as intensity-modulated radiotherapy (IMRT) (11) and image-guided radiotherapy (IGRT) (12). Modern IGRT methods for verification use daily online planar kilovoltage (KV) imaging and more recently, cone-beam CT (CBCT). Both methods show good agreement; however, CT provides additional information regarding bladder and rectal filling (13).

Radiopaque fiducials for prostate target localization are now widely used (14–17). Gold fiducials are most widely used as they have a high Z value, as a result they are highly visible with X-ray imaging. Gold fiducials, however, can cause artefacts on CT imaging which can interfere with contouring and verification. The ideal fiducial marker is easy to deliver with good visibility, minimal distortion on CT imaging, minimal dose perturbation, is biocompatible with soft tissue, and has a negligible risk of migration (18). It has been suggested that a polymer marker may be a better fiducial due to less imaging artefact (19).

We have previously characterized the gold and polymer fiducials in a uniform grey scale soft tissue equivalent phantom (20). Both fiducials were well seen as a dark hypointense marker on MRI but the gold fiducial produced greater artefact on CT based imaging. A clinical study was deemed appropriate before recommending the wide scale use of polymer fiducials in a prostate population. The aims of the study were to compare polymer fiducials to the standard gold fiducials using clinical radiotherapy protocols to assess the visibility and relative CT artefact production in a population of prostate cancer patients.

## Materials and methods

The study was a prospective investigation approved by the hospital ethics committee. Identical standard clinical prostate planning protocols were used for both fiducial types in terms of mode of fiducial insertion, imaging & simulation, treatment verification and image review assessment to provide a valid clinical assessment of the polymer fiducials. These protocols are outlined in the following text.

### Patient accrual

Patients with locally advanced prostate cancer with no contraindications to radiotherapy, MRI or fiducial insertion were invited to have polymer seeds inserted as part of a prospective study. Patients who wished to take part were entered into the study after giving their informed consent. Those patients who did not wish to participate were asked to join the comparator patient group, and underwent insertion of standard gold fiducials after completion of informed consent.

### Fiducials & insertion

Polymark™ (polymer) fiducials markers measuring 1mm x 3mm and Gold Soft tissue markers measuring 0.9mm x 3mm (CIVCO Medical Solutions, Kalona, Iowa, USA) were used. They were inserted into the peripheral prostate gland under sedation and antibiotic cover with transrectal ultrasound guidance. Each patient had a total of three fiducials implanted (one into the base, mid-gland and apex of the prostate).

### Imaging simulation

Patients were positioned supine with an individualized foam Alpha cradle placed on an indexed pelvic board with foot stocks. Patients were scanned on GE Lightspeed RT CT (Boston, Massachusetts, USA) 1.25mm slice width, helical, 0.75 pitch, no gap, 512x512 axial resolution, 650mm reconstruction diameter.

The planning MRI was performed on a 1.5 T Siemens Magneto Avanto Syngo MR B17^®^ (Siemens Healthcare, Erlangen, Germany). This study’s MRI sequence was a high-resolution T2-weighted scan with the following MRI parameters: T2 turbo-spin echo (TSE) with TR: 1250ms, TE: 185ms, slice thickness: 0.68mm, image matrix: 308x390 and FOV: 400x500mm with acquired voxel size of 1.30 (AP) x 1.28 (Lateral) x 1.35 (height) and reconstructed voxel size of 0.63 (AP) x 0.63 (Lateral) x 0.68 (height).

### Treatment verification

Patients were treated on Elekta Linacs (Stockholm, Sweden), using IMRT to a dose of 78Gy in 39 fractions over eight weeks. The departmental prostate verification protocol comprises daily pre-treatment, online cone-beam CT (CBCT). These were used to assess the visualization of the polymer and gold fiducials on CBCT. The standard CBCT parameters consisted of 41cm diameter FOV, variable M10/M20 (scan length 12 or 24cm) depending on target size, 120 kVp, 25 mA 40ms nominal per frame, 660 frames per scan (360 degrees rotation), 1mm voxel size, 2-3mm viewing slice resolution with an axial resolution of 512 x512.

Patients also underwent weekly 2D orthogonal KV planar imaging: anterior-posterior (AP) and lateral as part of the imaging study. The Elekta XVI (version 4.5+) KV imaging parameters were 120 kVp, 25 (AP) or 32 (lateral) mA and 40ms nominal per frame; five frames averaged per image, 25.6x25.6cm imaging area, 0.25mm nominal pixel size (Resolution 1024x1024).

### Image assessment

The planning CT and MR were transferred to MIM Maestro version 6.6.13 (Cleveland OH, USA) (MIM) as per clinical protocol for contouring. The visibility assessment and artefact analysis were performed in MIM.

All verification images were reviewed by the study radiation therapist, who routinely performs prostate radiotherapy verification. Three representative CBCT and three pairs of KV planar images (AP and Lateral) were selected for each patient for analysis at the start, mid and end of treatment. The CBCT and KV planar images were transferred to Mosaiq (Elekta, Stockholm, Sweden) for image review as per the centre’s standard verification process.

### Fiducial visibility

To measure the fiducial visibility and minimize inter-observer variability, the study radiation therapist scored all the images.

3D imaging typically shows a single fiducial being visible on a single slice. Therefore, each seed was scored for visibility. The visibility was scored for simulation CT & MR and verification CBCT on a scale 1 to 4 for each of the three fiducials apex, mid-gland and base as follows:

1. Clearly visible for verification
2. Visibility impaired but sufficient for verification
3. Visibility impaired but not sufficient for verification
4. Not visible and not sufficient for verification.

For 2D KV imaging, all three fiducials are visualized for verification simultaneously on each plane, i.e. AP and Lateral. Thus, the AP and lateral KV images were scored on the number of fiducials sufficiently visible for verification.

### Artefact analysis

A method was developed to analyze the artefact and seeds in a three-dimensional manner in the patient. A CT simulation represents the primary X-ray reference scan, therefore artefact due to the fiducials was measured on the planning CT quantitatively using MIM’s clinical imaging analysis tools.

The fiducials were initially manually contoured as for the standard verification. A 2cm diameter sphere was then created around the contour centre. The fiducial, bone, physiological calcifications, and rectal gas were subtracted from the sphere using the Boolean function to analyze the artefact’s impact on normal tissue in this sphere. The fiducials’ characteristics were separately investigated as they produced very high HU signals compared to the artefacts.

These spheres of interest contained the normal prostate tissues and the 3D artefact of the relevant fiducial seed. The voxel Hounsfield units (HU) for the sphere was exported in 5 HU bins for analysis and MIM was used to create histogram plots of HU versus voxel count from the spheres. These histograms were used to assess the relative differences in HU variation surrounding each of the fiducial markers.

Most voxels within the sphere are expected to be normal prostate tissue density. The bright and dark artefact is seen as the voxel variation of HU outside the normal tissue range at the extreme high and low HU values at either end of the histogram respectively. Therefore, the greater spread of the histogram, the less normal tissue is represented as it is hidden by the high and low HU artefact from the fiducial.

The spread of the histogram was analyzed in terms of the percentile ranges in HU. This range in voxel counts in the spherical contours (excluding the values attributable to each seed) was measured by considering percentile ranges of 1^st^ to 99^th^, 5^th^ to 95^th^, 10^th^ to 90^th^ and interquartile range (25^th^ to 75^th^).

Normal tissue which was not hidden by artefact was quantified by the proportion of voxels within normal tissue representative HU ranges of +/-100 HU and +/-150 HU. The greater the artefact, the less proportion of voxels were represented in this range.

### Statistical analysis

Frequencies and percentages were used to present results for categorical variables, with the Fisher’s (exact) test used to assess variability between groups. The mean ± standard deviation (SD) or median and inter-quartile ranges (IQR) were used to present continuous variables which were normally and non-normally distributed, respectively.

The percentile ranges and proportions were presented using median and IQRs and compared between the polymer and gold groups using the Mann-Whitney (rank-sum) test. Adjustment for the clustering of up to three fiducials within each patient was considered using a mixed-effects model, with independent covariance structure.

Patient imaging data were collected as per standard processes and stored within password-protected systems. Data were collected and prepared in Microsoft Excel and secured *via* password on a secure hospital server. Stata version 15.1 (College Station, Texas, USA) was used to conduct statistical analyses. A p-value of less than 0.05 was deemed to represent statistical significance.

## Results

### Patient cohort

Fourteen eligible patients with locally advanced prostate cancer were recruited to each arm. **Table 1** provides a comparison of the demographic characteristics between the two groups. The gold fiducial patients were slightly older than polymer patients (median age 77 vs 72 years; p=0.028), there were no statistically significant differences between the two groups. In relation to visibility of the fiducials on imaging, there was notably no significant difference in body mass index (BMI).

**Table 1:** Patient Characteristics across the two groups.

Factor	Gold	Polymer	^c^p-value
Number of Patients	14	14	
Age (years), median (^b^IQR)	77 (70, 78)	72 (68, 73)	0.028
^a^BMI, median (^b^IQR)	26.2 (24.7, 31.4)	27.7 (27.0, 29.9)	0.44
Gleason Score + Total			0.79
3 + 4 = 7	4 (29%)	6 (43%)	
4 + 3 = 7	3 (21%)	2 (14%)	
3 + 5 = 8	0 (0%)	1 (7%)	
4 + 4 = 8	3 (21%)	3 (21%)	
4 + 5 = 9	2 (14%)	2 (14%)	
5 + 4 = 9	2 (14%)	0 (0%)	
Initial PSA ug/L, median (^b^IQR)	16.5 (9.9, 27.0)	15.5 (7.2, 22.0)	0.41
Initial T staging			0.87
T1c	2 (14%)	4 (29%)	
T2a	2 (14%)	2 (14%)	
T2b	0 (0%)	1 (7%)	
T2c	2 (14%)	3 (21%)	
T3	2 (14%)	1 (7%)	
T3a	4 (29%)	2 (14%)	
T3b	2 (14%)	1 (7%)	

^a^BMI, Body mass index; ^b^IQR, interquartile range; ^c^p < 0.05 Statistical significance.

### Fiducial insertion

All patients had three fiducials successfully inserted without incident. One CT dataset and the KV images of two patients in the gold fiducial group and one CT dataset in the polymer fiducial group, could not be restored for analysis because of data corruption (**Tables 2**, **3**). Visibility results for 3D imaging are shown in **Table 2**.

**Table 2:** MRI, CT and CBCT fiducials visibility assessments.

Fiducials and ^a^Visibility Grade	MRI Visibility			CT Visibility			CBCT Visibility		
	Gold	Polymer	^c^p-value	Gold	Polymer	^c^p-value	Gold	Polymer	^c^p-value
Number of Patients	14	14		14	14		14	14	
**Apex fiducial**			1.00			<0.001			0.22
1	0	1 (7%)		1 (7%)	12 (86%)		14 (100%)	11 (79%)	
2	2 (14%)	1 (7%)		9 (64%)	2 (14%)		0	1 (7%)	
3	2 (14%)	2 (14%)		3 (21%)	0		0	2 (14%)	
4	10 (71%)	10 (71%)		0	0		0	0	
†Not restored	0	0		1 (7%)	0		0	0	
**Mid Gland fiducial**			1.00			<0.001			0.22
1	0	1 (7%)		1 (7%)	9 (64%)		14 (100%)	11 (79%)	
2	2 (14%)	1 (7%)		7 (50%)	5 (36%)		0	3 (21%)	
3	3 (21%)	2 (14%)		5 (36%)	0		0	0	
4	9 (64%)	10 (71%)		0	0		0	0	
†Not restored	0	0		1 (7%)	0		0	0	
**Base fiducial**			0.63			<0.001			0.098
1	0	2 (14%)		0	13 (93%)		14 (100%)	10 (71%)	
2	2 (14%)	1 (7%)		8 (57%)	1 (7%)		0	4 (29%)	
3	2 (14%)	2 (14%)		5 (36%)	0		0	0	
4	10 (71%)	9 (64%)		0	0		0	0	
^b^Not restored	0	0		1 (7%)	0		0	0	

a Fiducials were scored for visibility i.e.

1. Clearly visible for verification.

2. Visibility impaired but sufficient for verification.

3. Visibility impaired but not sufficient for verification.

4. Not visible and not sufficient for verification.

b Not restored refers to images lost due to data corruption.

c p < 0.05 Statistical significance.

**Table 3:** 2D KV Visibility assessments.

KV Type and Number of Fiducials Visible	Gold	Polymer	^d^p-value
Number of Patients	14	14	
^a^LATKV, Start of Radiotherapy No. fiducials visible			<0.001
0	2 (14%)	12 (86%)	
1	0	1 (7%)	
2	5 (36%)	0	
3	5 (36%)	0	
†Not restored	2 (14%)	1 (7%)	
^a^LATKV, Mid Radiotherapy No. fiducials visible			<0.001
0	1 (7%)	14 (100%)	
1	2 (14%)	0	
2	4 (29%)	0	
3	5 (36%)	0	
† Not restored	2 (14%)	0	
^a^LATKV, End of Radiotherapy No. fiducials visible			<0.001
0	1 (7%)	13 (93%)	
1	0	0	
2	6 (43%)	0	
3	5 (36%)	0	
Not restored	2 (14%)	1 (7%)	
^b^APKV Start of Radiotherapy, No. fiducials visible			0.14
0	0	0	
1	0	3 (21%)	
2	2 (14%)	3 (21%)	
3	10 (71%)	8 (57%)	
†Not restored	2 (14%)	0	
^b^APKV Mid Radiotherapy No. fiducials visible			0.65
0	0	1 (7%)	
1	0	1 (7%)	
2	2 (14%)	3 (21%)	
3	10 (71%)	9 (64%)	
Not restored	2 (14%)	0	
^b^APKV End of Radiotherapy No. fiducials visible			1.00
0	0	1 (7%)	
1	0	0	
2	2 (14%)	3 (21%)	
3	10 (71%)	9 (64%)	
^c^Not restored	2 (14%)	1 (7%)	

^a^LATKV, Lateral KV planar image; ^b^APKV, Anterior Posterior KV planar image; ^c^Not restored refers to images lost due to data corruption, ^d^p < 0.05 Statistical significance.

### Fiducial visibility

**Figure 1:**
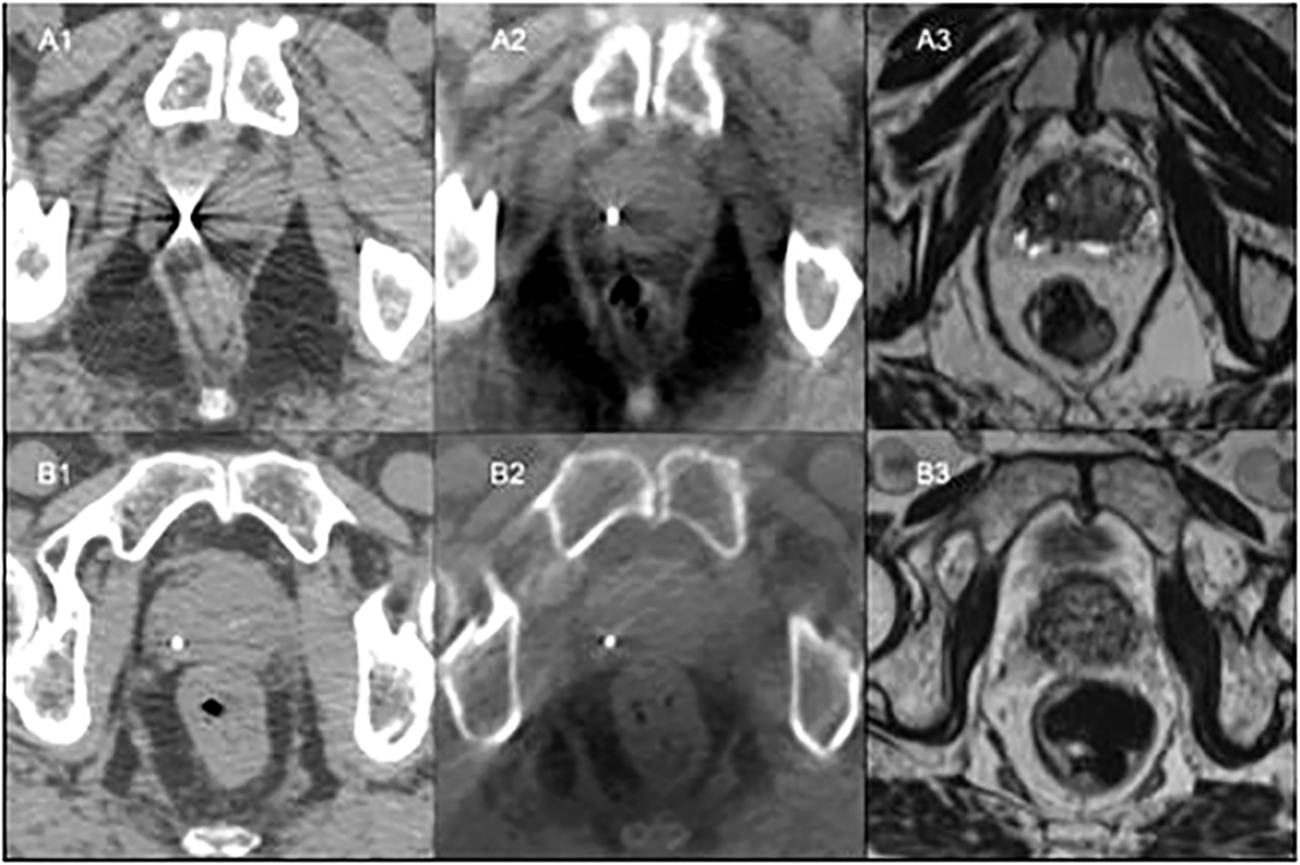
Patient A: **(A1)** - CT simulation with gold fiducial with associated artefact on CT. **(A2)** - Fiducial co-registered CBCT showing gold fiducial with little artefact. **(A3)** - Bone co-registered MRI with gold fiducial not visible. Patient B: **(B1)** - CT simulation showing polymer fiducial with minor artefact. **(B2)** - Fiducial co-registered CBCT with visible polymer fiducial with slight artefact. **(B3)** - Bone co-registered MRI with polymer fiducial not visible.

The gold fiducials were sufficiently visible with CT (**Figure 1A1**) for verification in 71%, 57% and 57% of those positioned in the apex, mid-gland and base, respectively. However, the polymer fiducials scored significantly higher in terms of visibility with CT imaging (p<0.001) due to less artefact and were visible for verification in 100% of patients (**Figure 1B1**).

Both the gold fiducials and polymer fiducials were well seen on CBCT, with no significant differences in visibility scores. However, all the gold fiducials (100%) scored 1 (clearly visible for verification) compared to 71-79% of the polymer fiducials. As anticipated, the gold fiducials were clearly seen, with less prominent artefact on CBCT than CT (**Figures 1A2, B2**).

The visibility of the gold and polymer fiducials for MRI was low (**Figures 1A3, B3**). In 64-71% of the images, neither the gold nor the polymer fiducials images could be seen on MRI.

Gold markers were well visualized on KV imaging. (**Table 3**). At least two of the gold fiducials were seen on 65-79% of the lateral KV images and 85% of the AP KV images. In contrast, only one polymer fiducial was visualized on lateral KV images across the three-time points due to the overlying pelvic bones. Polymer fiducials were better seen on the AP KV images, with at least two fiducials visualized in 78-85% of the patient images.

### CT fiducial and artefact analysis

The fiducial contours had a small volume and low voxel count, consequently only the maximum HU was considered as it is most representative of the fiducial material. The polymer fiducial contours and gold fiducial contours had similar volume and voxel counts. The polymer fiducial contours’ median volume was 0.03cc (range 0.01 to 0.07cc) compared to 0.03 cc (range 0.01 to 0.11 cc) for gold. The polymer group’s voxel count had a median of 15 (range 5 to 34) voxels as did the gold fiducials group with a median of 15 (range 5 to 52) voxels. However, the maximum HU value for polymer was lower, with a median of 2603 (range 1564 to 3350) HU compared to 18017 (range 3025 to 23635) HU for gold. The gold fiducials had a much higher HU contrast, i.e. brighter than polymer, on CT (**Figures 1A1, B1**).

**Figure 2:**
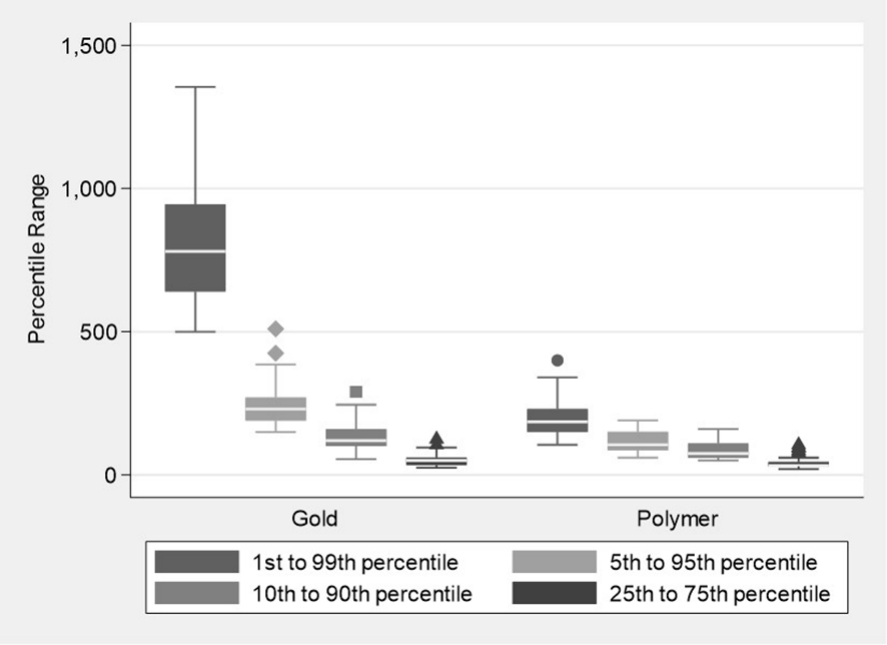
Boxplots showing the distribution of percentile ranges in the Hounsfield units for the Polymer and Gold groups.

Box plots of the distribution of the percentile ranges (HU) (**Figure 2**) illustrate the range in voxel counts across the artefact spheres’ HU range. It is a graphical representation of the relative width of the histogram. The plots showed a significantly lower median voxel count across the HU range for the polymer fiducials compared to gold fiducials. The median HU values (IQR) polymer vs gold were 185 (150 to 230) vs 780 (640 to 945) (p<0.001) for the 1^st^ to 99^th^ percentile; 105 (85 to 150) vs 230 (190 to 270) (p<0.001) for the 5^th^ to 95^th^ percentile; 75 (60 to 110) vs 120 (100 to 160) (p<0.001) for the 10^th^ to 90^th^ percentile and 33 (39 to 45) vs 50 (35 to 60) (p=0.002) for the 25^th^ to 75^th^ percentile respectively (**Figure 2**). The polymer values show fewer voxels at the extreme HU artefact values and a narrower histogram than gold, suggesting that the polymer fiducials produced fewer artefacts on CT images.

**Figure 3:**
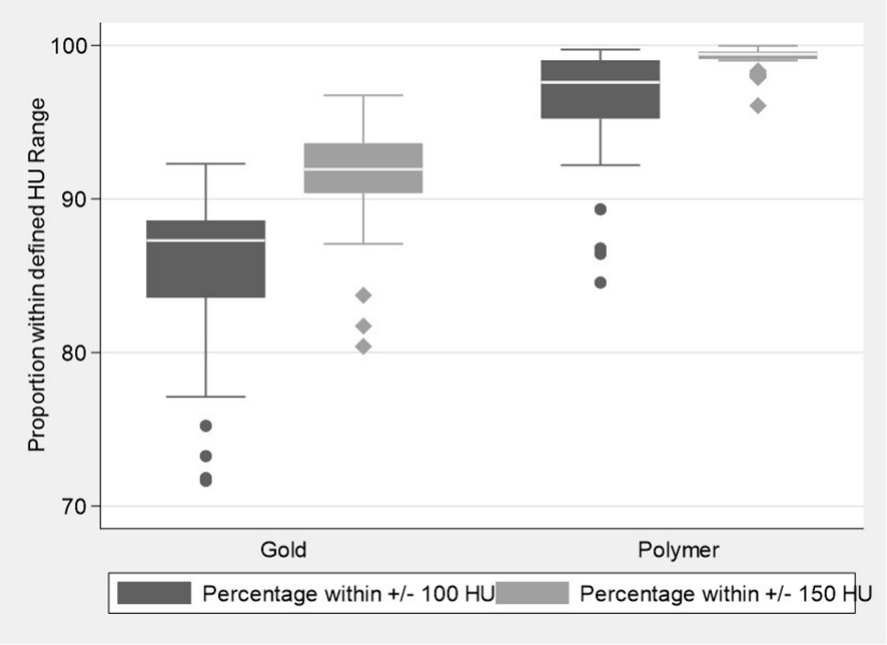
Boxplots illustrating the distribution of the proportion of voxel counts within the defined Hounsfield unit normal tissue ranges for the Polymer and Gold groups.

The proportion of voxel counts within the pre-defined HU normal tissue ranges of ±100 and ±150 HU, (**Figure 3**) were also significantly higher for the polymer fiducials when compared to the gold fiducials (p values <0.001), with a median (IQR) of 97.6% (95.23, 99.04) in the polymer group compared to 87.3% (83.56, 88.60) for the gold group when considering the ±100 HU defined range and 99.4% (99.12, 99.60) compared to 91.9% (90.39, 93.61) for the ±150 HU range. These results show less prostate was obscured by artefact as a significantly greater proportion of prostate normal tissue was visualized for the polymer fiducials compared to gold. These statistically significant differences were maintained upon mixed-effects modeling, with the cluster effect of up to three fiducials per patient taken into account (all p-values <0.001).

## Discussion

The study is one of the first to examine and validate the use of polymer fiducials in a prostate cancer patient population. It found that while gold fiducials have a higher HU and are therefore brighter; they produce far more artefact than polymer fiducials on CT. The reduction in artefact around the polymer fiducials resulted in a greater volume of normal tissue being visualized as it was not obscured by high and low HU artefact seen with the gold. Both the polymer and gold fiducials were well seen on CBCT. However, the lower HU of the polymer fiducials compared with gold meant they could not be easily detected on lateral KV imaging as they were obscured by the pelvic bones. During the clinical optimization process, it was noted that the polymer fiducials could be seen on oblique KV images if bone was minimized but increasing KV for the lateral images did not sufficiently improve their visibility. Neither type of fiducial could be seen on 1.5T T2 MRI in contrast to phantom studies (21) and they were therefore not useful for CT to T2 MRI co-registration.

The accurate localization of the prostate is crucial for precise radiotherapy - imaging can be used to localize the target and reduce uncertainty during treatment. IGRT can significantly minimize patient setup uncertainties and achieve better conformal radiation therapy (22). 3D multi-planar and multi-modality imaging is routinely used for contouring, but 3D imaging is now increasingly utilized for verification. Verification is mainly performed with CBCT, although MRI is being investigated (23).

IGRT historically used pelvic bony anatomy to assess patient alignment. However, the prostate can move relative to the pelvic bones, and thus bony alignment is considered insufficient for dose-escalated IMRT for prostate cancer (14–17). A more accurate prostate target localization method uses biologically inert radiopaque fiducial markers (usually gold as it is well visualized on X-ray imaging). Typically, three fiducials are inserted into the prostate, i.e. apex, mid-gland and base, for better accuracy and reproducibility of prostate alignment. Three fiducials also reduce localization uncertainty due to migration of the fiducials and deformation of the prostate (16, 24, 25).

Comparison studies of IMRT with IGRT using fiducial markers versus non IGRT treatments have shown a decrease in late gastrointestinal and genitourinary toxicity (12, 26, 27) and in one study there was a significant improvement in prostate cancer outcome (12). The toxicity difference can be attributed to the IMRT technique’s combination of more conformation dose distribution leading to reduced dose to organs-at-risk as well as daily image guidance, which permits safe reduction of PTV margins.

The shortcomings of gold fiducials are that they cause bright radiating and dark shadowing artefact on CT imaging leading to a change in perceived target and normal tissue density. The distorted CT image can also result in inaccurate delivery if not accounted for (28). The artefacts can also hide anatomical detail that could lead to inaccurate contouring. The obscuring of anatomical detail is most common around the apex of the prostate. The artefact can also interfere with the efficiency and accuracy of verification. Polymer fiducials appear to alleviate these issues with CT.

The limitation of the study is that we compared only polymer fiducials to the standard gold fiducials. Other new fiducial markers produce minimal distortion with CT imaging. Visicoil uses helical gold coils to reduce the relative thickness and decrease the equivalent density, thus lessening the image artefact (29). Others use a mixture of low-density biocompatible materials and gold particles (18) or lower Z radiopaque materials such as stainless steel, titanium (30) carbon or ceramic substances (16, 19). A study of gold, carbon and polymer fiducials showed that all fiducials could be identified on the CT and KV images in a phantom (19) and the findings regarding the gold and polymer fiducials are similar to the preliminary phantom (20) and present study. I.e., Gold fiducials demonstrated the highest contrast but had significant artifact on CT, while minimal or no artifacts were observed with carbon and polymer fiducials, respectively (19).

The present study showed that neither fiducial could be adequately visualized for accurate co-registration or verification when using 1.5T T2 weighted MRI. However, they are clearly visible in uniform gray scale phantoms when using T1 3T and 1.5T MRI (21) and 1.5T T2 MRI (20) as a hypointense dark focus. Thus, it is likely that both fiducials appear as a hypointense focus clinically within the prostate. But they are difficult to discern from the multiple physiological hypointense foci found in a patient’s heterogeneous gray scale prostate.

In phantom studies, gold markers are better seen on T1 weighted 3T MRI than T1 1.5T (21). Our clinical experience with diagnostic 3T MRI T1 and T2 sequences did not improve the differentiation of gold fiducials from other hypointense foci within the prostate. The issue is not so much resolution but the heterogeneous gray scale of the prostate as opposed to the uniform gray scale of phantoms. A limitation of the study is that we did not investigate other sequences that better visualize and differentiate gold fiducials on MRI such as T2*2D & T2*3D (30) and multi-parametric MRI with bTFE (balanced steady-state free precession sequence) (31, 32). However, these are not yet used widely because of limited resources. Further investigations into these imaging variables are in progress. Furthermore, we did not investigate artefact suppression CT (28) as it was not available at our center at the time of the study.

## Conclusion

In conclusion, this patient study has shown that polymer fiducials are preferable to gold fiducials for CT and CBCT in prostate cancer patients because of minimal artefact and good visibility. Polymer fiducials have minimal distortion with CT imaging, minimal dose perturbation, and are biocompatible with soft tissue. However, it was not well seen on T2 weighted MRI or KV imaging and therefore not suitable for image co-registration or 2D planar KV verification including real time intra-fractional motion monitoring.

## Data availability statement

The raw data supporting the conclusions of this article will be made available by the authors, without undue reservation.

## Ethics statement

The study was fully ethically approved by the Hospital Health Human Research Ethics Committee, Project No: H2013/04976. The patients/participants provided their written informed consent to participate in this study.

## Author contributions

DL - Concept, data collection and analysis, interpretation, manuscript. CB - Input into data collection, interpretation, manuscript. BH - Input into data collection, analysis and interpretation. MT - Input into data analysis and interpretation. DS - Input into concept, data collection & interpretation. NL - Input into data collection/acquisition. MS - Input into manuscript and interpretation. OF - Input into manuscript, editing and revision. MH - Input into data collection/acquisition. MC - Input into manuscript and interpretation. FF - Input into manuscript and interpretation. TJ - Input into manuscript and interpretation. DA - Input into data collection/acquisition. MW - Input into manuscript and interpretation. SS - Input into data collection/acquisition. VK - Input into manuscript and interpretation. All authors contributed to the article and approved the submitted version.
